# Clinique de l'Hôpital Saint-Louis; ou, Traité complet des Maladies de la Peau

**Published:** 1833-10

**Authors:** 


*Clinique de l'Hôpital Saint-Louis; ou, Traité complet des Maladies de la Peau: contenant la Description de ces Maladies, et leurs meilleurs Modes de Traitement. Orné de* 63 *Planches gravées au burin, parfaitement coloriées, et retouchées au pinceau.* Par M. le Baron J. L. ALIBERT, Officier de la Legion d'Honneur, Chevalier de plusieurs Ordres, Médecin en chef de l'Hôpital Saint-Louis, &c. 4me Livraison. 'A Paris, 1832.

THIS is the second edition of the celebrated work of Alibert on the diseases of the skin. An additional experience of more than twenty years has enabled him to arrange his materials in better order, and he can no longer be charged, as he was by Dr. Bateman, with a "total defect of discrimination and method." It must be confessed, however, that Alibert is weak in the diagnosis: in the present number, which contains the *Dermatoses dartreuses,* he gives no distinct definition of this group, and is contented with endeavouring to distinguish it from the last one, the *Dartres teigneuses*. Even Bateman, however, allowed that Alibert was fortunate in the artists whom he employed; and it must be granted, that he is as superior in pictorial illustration as our countryman was in nosological definition. In the present number, the Herpès furfureux circiné, and the Melitagre chronique ou nigricante, are exquisite specimens both of drawing and colouring. Their breadth, freedom, and bold relief, are such as are rarely met with in the illustrations of medical works. This group, the *dartreuses*, is divided into four genera, Herpes, Varus, Melitagra, and Esthiomenos. The Herpes of Alibert is equivalent to the Psoriasis of Bateman: thus, the plate which he mentions as giving a good representation of Psoriasis palmaria illustrates what Alibert calls Herpès squameux centrifuge, (the Dartre squameuse centrifuge of the former edition.) The Varus of our author is the Acne of Willan or Bateman. He observes, that varus is a Latin name for this disease, and occurs in the old pleasantry, a sort of Roman Joe-Miller: "*Miramur cur Servilius pater tuus, homo constantissimus, te nobis tam varium reliquerit.*"

Under the name of *Varus orgéolé*, or *Varus hòrdeolatus*, he speaks of a chronic hordeolum, or stye, which he says must not be confounded with the acne furuncule that may arise in the same situation: the *varus* may last a lifetime. Alibert thinks that *lippus*, in Horace, means afflicted with this chronic stye. On this point we rather side with the multitude, who suppose lippus to mean sore-eyed; in other words, affected with a slight ophthalmia.

Another species of varus is the *Varus goutte-rose*, the Gutta rosacea of many books, the Acne rosacea of Bateman, who observes: "This species of Acne seldom occurs in early life, except where there is a great hereditary predisposition to it: in general it does not appear before the age of forty; but it may be produced in any person by the constant immoderate use of wine and spirituous liquors. The greater part of the face, even the forehead and chin, are often affected in these cases; but the nose especially becomes tumid, and of a fiery red colour; and, in advanced life, it sometimes enlarges to an enormous size, the nostrils being distended and patulous, or the alæ fissured, as it were, and divided into several separate lobes." And Sennert, who is quoted by Bateman, saw a man whose nose had attained such a size as to be a hindrance to him in reading; for which reason he had some slices cut off, in the year 1629. (*Pract. Med.*, lib. v. part i. cap. 31.)

Alibert confesses that, in these desperate cases, cosmetics are in vain: "cet accident est fréquent chez les femmes, et il se montre souvent irréparable. On peut sans doute a l'aide d'un fard plus ou moins ingénieusement inventé, dissimuler, chez elles, les ravages occasionnés par le temps, corriger des teintes défectueuses, effacer jusqu'aux traces d'une légère altération cutanée; mais les prestiges et les soins étudiés de la coquetterie la plus raffinée ne sauraient corriger ces engorgemens partiels qui se forment dans l'épaisseur des tégumens, qui changent les rapports, et la configuration des traits, qui enlèvent à la physiognomie sa régularité, sa finesse, et son charme."

The third genus, or Melitagra, (the Impetigo of Willan and Bateman,) is divided into two species only, the acute and chronic. The following case, which is an example of the acute form, was treated in the hospital of St. Louis:

"A woman, aged thirty, of a bilious temperament, black hair, and a dark but very fine skin, observed, during the hot summer weather, that a small acuminated pustule had arisen above the right eyelid; its base was surrounded by a deep red areola. New pustules began to appear on different parts of her face, forming by their union a kind of corymb. The sero-purulent fluid contained in them soon burst forth, but, hardly had it been exposed to the contact of the atmosphere, when it coagulated in round crusts of a bright yellow, whose colour might be very properly compared to new honey, or to the globules of gum which in spring transude through the bark of plum-trees. These patches appeared to be the result of a real crystallisation. When carefully examined, each of them was seen to be formed by the junction of small cylinders, which were pointed where they emerged from the skin, and globular at the other end.* Each patch contained as many of them as there were pustules, and hence the surface of the crust was wrinkled and irregular. The crusts were surrounded by a bright red areola, which did not extend more than two lines at most; the rest of the intervening skin was perfectly healthy. Both eyelids, which were of a lively red, were covered with yellow patches, and both eyes were affected with a slight ophthalmia. The lobe of each ear was also covered with honey-like patches, not so thick in consistence, and of a greenish-yellow colour. When these patches were moistened with tepid water, they were partly dissolved, and were easily detached. This singular property of being soluble in water is a fresh reason for comparing these morbid products to gummy substances. (*Alibert*, page 184.)"

Two cases of Melitagra were cured by ioduro-sulphureous lotions; and Alibert remarks, that this shows why warm

* The baron should have called these *pointed cylinders* inverted cones.—ED.

sulphureous baths, containing traces of iodine, are so efficacious in the majority of cutaneous diseases.

The fourth genus, or Esthiomenos, is the Lupus of Willan, Bateman, and Plumbe. Miners, braziers, curriers, tanners, &c. are very liable to this horrible disease, if they neglect the hygienic precautions which their trades require. The Esthiomenos is a disease of youth, and disappears with the approach of manhood; but the unfortunate patients are so disfigured by it as to be disqualified for mixing in society, and therefore cannot resume their former occupations; they usually therefore take the place of servant in some hospital, or other charitable institution. The Esthiomenos is not to be confounded with the Noli me tangere, which is a disease of manhood and advanced life.

Some of our readers will be glad to hear that the price of this second edition of Alibert's great work is exactly half the price of the first; being three hundred francs instead of six hundred.

